# *Curcuma longa* extract reduces serum inflammatory markers and postprandial hyperglycemia in healthy but borderline participants with overweight and glycemia in the normal/prediabetes range: a randomized, double-blind, and placebo-controlled trial

**DOI:** 10.3389/fnut.2024.1324196

**Published:** 2024-01-29

**Authors:** Ryusei Uchio, Chinatsu Okuda-Hanafusa, Haruka Sakaguchi, Ryosuke Saji, Koutarou Muroyama, Shinji Murosaki, Yoshihiro Yamamoto, Yoshitaka Hirose

**Affiliations:** Research & Development Institute, House Wellness Foods Corp., Itami, Hyogo, Japan

**Keywords:** *Curcuma longa* (turmeric) extract, bisacurone, turmeronol, chronic inflammation, C-reactive protein, hemoglobin A1c, insulin sensitivity, oral glucose tolerance test

## Abstract

The spice turmeric, which has the Latin name *Curcuma longa* (*C. longa*), has various physiological effects. This study evaluated the effects of a hot water mixture with supercritical carbon dioxide *C. longa* extracts, CLE, and the potential active components of *C. longa*, turmeronols A and B and bisacurone on inflammation and glucose metabolism. First, we investigated the effect of CLE and the potential active components of *C. longa* on lipopolysaccharide-induced inflammation in RAW264.7 macrophages. We found a significant decrease in the production of interleukin (IL)-1β, IL-6, tumor necrosis factor (TNF)-α, and nitric oxide with CLE, turmeronol A, and bisacurone, Significant inhibition of each of these substances was also observed, except for TNF-α with turmeronol B. The second part of our work was a 12-week randomized, double-blind, placebo-controlled study in healthy but borderline adults aged 40 to 69 years with overweight and normal/prediabetes glycemia. We compared blood inflammatory and glycometabolic markers in the CLE (*n* = 55) and placebo groups (*n* = 55). We found significantly lower serum high-sensitivity C-reactive protein and hemoglobin A1c levels in the CLE group. This group also showed significant improvements in postprandial hyperglycemia and insulin sensitivity indices. Our findings indicate that CLE may reduce low-grade inflammation and thus improve insulin sensitivity and postprandial hyperglycemia.

**Clinical trial registration:** https://center6.umin.ac.jp/cgi-open-bin/ctr/ctr_view.cgi?recptno=R000051492, UMIN-CTR, UMIN000045106.

## 1 Introduction

Acute inflammation is a physiological process for eliminating pathogens and dead cells and induces the repair and regeneration of damaged tissue. In contrast, chronic inflammation is an inflammatory response that is low-grade, persistent, and mediated by long-lived immune cells such as macrophages (1). The latter type of inflammation is induced by obesity (2), aging (3), and unhealthy lifestyle factors, such as unbalanced diet, physical inactivity, insufficient sleep, and psychological stress (1, 4, 5). Macrophages play a key role in the development of chronic inflammation by producing inflammatory mediators that induce cell damage, tissue infiltration of inflammatory cells, and impairment of systemic metabolisms such as glycometabolism (1, 6). Clinical human studies generally use C-reactive protein (CRP) as a marker of low-grade, systemic inflammation (5, 7), and previous studies have shown that mildly elevated CRP is associated with an increased risk of metabolic syndrome (8), diabetes (9), impaired cognitive function (10), coronary heart disease (11), and cancer (12).

Glucose is one of the energy sources for mammalian cells and has an important role in maintaining normal physiological homeostasis in humans (13). High systemic glucose levels are reduced by insulin, which is released from the pancreas and promotes glucose uptake in insulin-sensitive peripheral tissues, such as skeletal muscles, and inhibits hepatic gluconeogenesis (14). Excessive intake of nutrients such as carbohydrate and fat causes obesity through accumulation of visceral fat, which contributes to abnormal glycometabolism (14). One method for evaluating glucose tolerance, insulin secretory capacity, and insulin sensitivity is the oral glucose tolerance test (OGTT), which measures blood levels of glucose and insulin after oral administration of 75 g of glucose. The OGTT is also widely used to diagnose impaired glucose tolerance (prediabetes) and type 2 diabetes. As a marker of early impaired glucose homeostasis, the post-load glucose level in the OGTT is a more sensitive marker of prediabetes than fasting blood glucose and hemoglobin A1c (HbA1c) (15). Individuals with overweight or obesity have been reported to show an increased number of activated macrophages, which produce inflammatory mediators that can inhibit the action of insulin (6, 16). These chronic inflammatory states contribute to the induction of impaired insulin sensitivity (also called insulin resistance) and the development of type 2 diabetes (1, 5). In previous clinical studies, participants with overweight and low-grade inflammation exhibited a significant increase in glucose plasma levels in the OGTT (17).

The spice turmeric, which has the Latin name *Curcuma longa* (*C. longa*), belongs to the Zingiberaceae family and exerts a range of physiological effects (18). Curcumin, a lipophilic polyphenol, is a major component of *C. longa* and has various physiological activities, including anti-oxidant, anti-inflammation, and anti-obesity actions (19, 20). Curcumin also prevents the development of endothelial dysfunction and atherosclerosis (20). Curcumin-free *C. longa* water extracts have also been reported to exhibit numerous activities (21). Other components of *C. longa* include the sesquiterpenoids turmeronols A and B and bisacurone (22, 23). Turmeronols are known to inhibit the production of inflammatory mediators in immune cell lines by inhibiting the signaling pathway of nuclear factor kappa-light-chain-enhancer of activated B cells (NF-kB) (24). Bisacurone inhibits adhesion of monocytes to endothelial cells through down-regulation of vascular cell adhesion molecule 1 (VCAM-1) protein expression (25). In addition, water extracts of *C. longa* as well as bisacurone were reported to inhibit ethanol-induced liver injury (26). In diabetic animal models, bisacurone was found to reduce systemic glucose levels and suppress cardiotoxicity and nephropathy (27). *C. longa* water extracts are known to exert anti-oxidant and anti-inflammatory effects (28–30), prevent atrophy of skeletal muscle (31), increase dermal water content (32), reduce fatigue (33), and improve depression (34). In animal disease models, *C. longa* water extracts decreased expression of inflammatory cytokine and chemokine mRNA and prevented chronic inflammatory diseases, e.g., carbon tetrachloride-induced hepatitis (35) and non-alcoholic steatohepatitis (36). Also, in animal models, supercritical carbon dioxide *C. longa* extracts suppressed inflammation induced by carrageenan (37). A recent study using streptozotocin-induced diabetic rats demonstrated that *C. longa* extracts improved postprandial glucose levels (38). In addition, daily intake of a mixture of hot water and supercritical carbon dioxide *C. longa* extracts (CLE) improved mental health-related quality of life questionnaire scores (22, 23). However, to date, the effects of CLE on chronic inflammatory processes and postprandial glucose metabolism in humans remain unclear.

Therefore, to investigate whether CLE and its potential active ingredients turmeronols A and B and bisacurone have anti-inflammatory activities, we first conducted a non-clinical study in which we assessed their effects on the production of inflammatory mediators in macrophage cells stimulated with lipopolysaccharide (LPS). Subsequently, to evaluate the effect of CLE on chronic inflammation and postprandial glycometabolism, we conducted a randomized, double-blind, placebo-controlled trial to measure the levels of inflammatory markers and glycometabolic markers in male and female participants with overweight and glycemia in the normal/prediabetes range.

## 2 Materials and methods

### 2.1 Non-clinical study

#### 2.1.1 Reagents

We used the following reagents: Dulbecco’s modified Eagle’s medium (DMEM), penicillin–streptomycin, LPS (*Escherichia coli* O127:B8; L3129), and dimethyl sulfoxide (Sigma-Aldrich, Saint Louis, MO, USA); fetal bovine serum (FBS) (HyClone, Logan, UT, USA); turmeronols A and B and bisacurone isolated from *C. longa* (Nagara Science, Gifu, Japan); and sulfanilamide, N-1-naphthylenediamide dihydrochloride, phosphoric acid, and sodium nitrite (Fujifilm Wako, Osaka, Japan).

#### 2.1.2 Preparation of CLE

**Figure 1:**
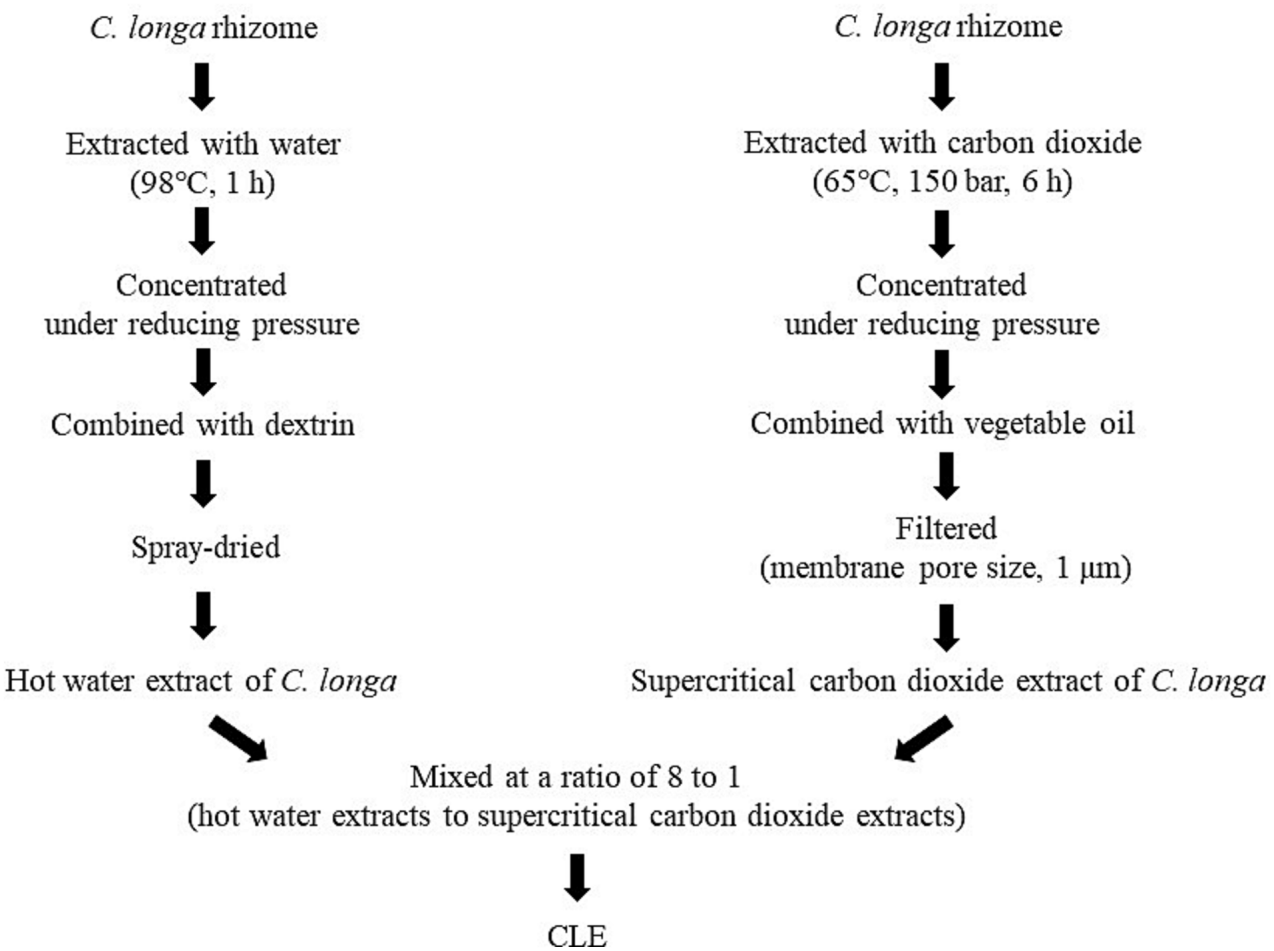
Preparation flow diagram of CLE. CLE, a mixture of a hot water extract and a supercritical carbon dioxide extract of *Curcuma longa.*

CLE (Turmeric Extract Mixture, House Wellness Foods) was produced as described elsewhere (22, 28, 39). In brief, for the hot water extract, *C. longa* rhizomes were placed in water at 98°C for 1 h, then the concentrated supernatant was combined with dextrin and spray dried. The supercritical carbon dioxide extract was obtained by placing rhizomes in carbon dioxide at 65°C at a pressure of 150 bar for 6 h. Then, the supernatant was concentrated, combined with vegetable oil, and filtered (membrane pore size, 1 μm). These two *C. longa* extracts were mixed at a ratio of 8 to 1 (the hot water extracts to the supercritical carbon dioxide extracts) and the mixture was used as CLE (Figure 1). The safety of CLE was confirmed in a previous clinical intervention study in which participants consumed excess doses of CLE (10 capsules per day, i.e., a 5-fold higher dose than in this study). The previous study found no adverse effects of the intervention on physical measurements, hematology and biochemistry tests, or urinalysis (23).

#### 2.1.3 Measurement of turmeronols and bisacurone in CLE

CLE was dissolved in ethyl acetate, vigorously mixed with an equal volume of distilled water, and centrifuged at 400 × g for 5 min at room temperature. The ethyl acetate layer was collected and evaporated to dryness under nitrogen to remove the ethyl acetate. The residue was dissolved in methanol and then filtered and injected into an HPLC system under the following conditions. Turmeronol A: Separation was carried out on an octadecylsilyl (ODS) column (Chemical Evaluation Research Institute, Tokyo, Japan) with a mobile phase consisting of 0.1% formic acid-acetonitrile (52:48, v/v), and the derivative was detected at 238 nm. Turmeronol B: Separation was performed on a pyrenylethyl column (Nacalai Tesque Inc., Kyoto, Japan) with a mobile phase composed of distilled water–methanol (43:57, v/v), and the derivative was detected at 238 nm. Bisacurone: Separation was performed on an ODS column (FUJIFILM Wako Pure Chemical Corp., Osaka, Japan) with a mobile phase consisting of acetonitrile-0.005% trifluoroacetic acid (30,70, v/v), and the derivative was detected at 240 nm.

#### 2.1.4 Cell culture

To create the cell line, RAW264.7 mouse macrophages (RIKEN BioResource Center, Tokyo, Japan) were seeded on 96-well plates (1.5 × 10^5^ cells/well) and incubated for 24 h in 150 μL of DMEM with 10% FBS, 100 U/mL penicillin, and 100 μg/mL streptomycin at 37°C with 5% carbon dioxide. The cells were separated from the medium and preincubated for 1 h in FBS-free DMEM containing 600 μg/mL of CLE or turmeronol A, turmeronol B, or bisacurone; the concentrations of these potential active ingredients were determined beforehand and were equivalent to those naturally found in CLE, as follows: turmeronols A and B, both 1.1 μM, and bisacurone, 4.2 μM. In previous animal and clinical studies, the markers of inflammation and glycometabolism were improved by various plant extracts, including *Houttuynia cordata* Thunb. and *Morus alba* L. (40–43). These extracts significantly inhibited the production of inflammatory mediators at approximately 300–800 μg/mL in Raw264.7 cells (41, 44). The concentration of CLE was determined to be 600 μg/mL by conducting preliminary experiments based on these reports. Preliminary experiments had confirmed that none of the potential active components of CLE were cytotoxic (24, 45). In the last step, the cells were stimulated with LPS (final concentration, 200 ng/mL) for 15 h, and inflammatory factors were assessed in culture supernatants.

#### 2.1.5 Measurement of inflammatory mediators

We used DuoSet enzyme-linked immunosorbent assay (ELISA) kits for mouse interleukin (IL)-1β, IL-6, and tumor necrosis factor (TNF)-α (R&D Systems, Rochester, MN, USA) to determine the concentrations of these inflammatory mediators. Procedures were performed according to the manufacturer’s instructions. In addition, we applied the Griess method to assess nitrite, a stable metabolite of nitric oxide (NO) (46, 47).

#### 2.1.6 Statistical analysis

We used one-way analysis of variance (ANOVA) and Dunnett’s multiple comparison test to compare LPS-stimulated control and treated cells. Results are presented as means and standard deviation (SD). The statistical significance level was set at 0.05. Statcel 4 software (OMS Publishing, Tokorozawa, Japan) was used for statistical analysis.

### 2.2 Clinical intervention study

#### 2.2.1 Study design

**Figure 2:**
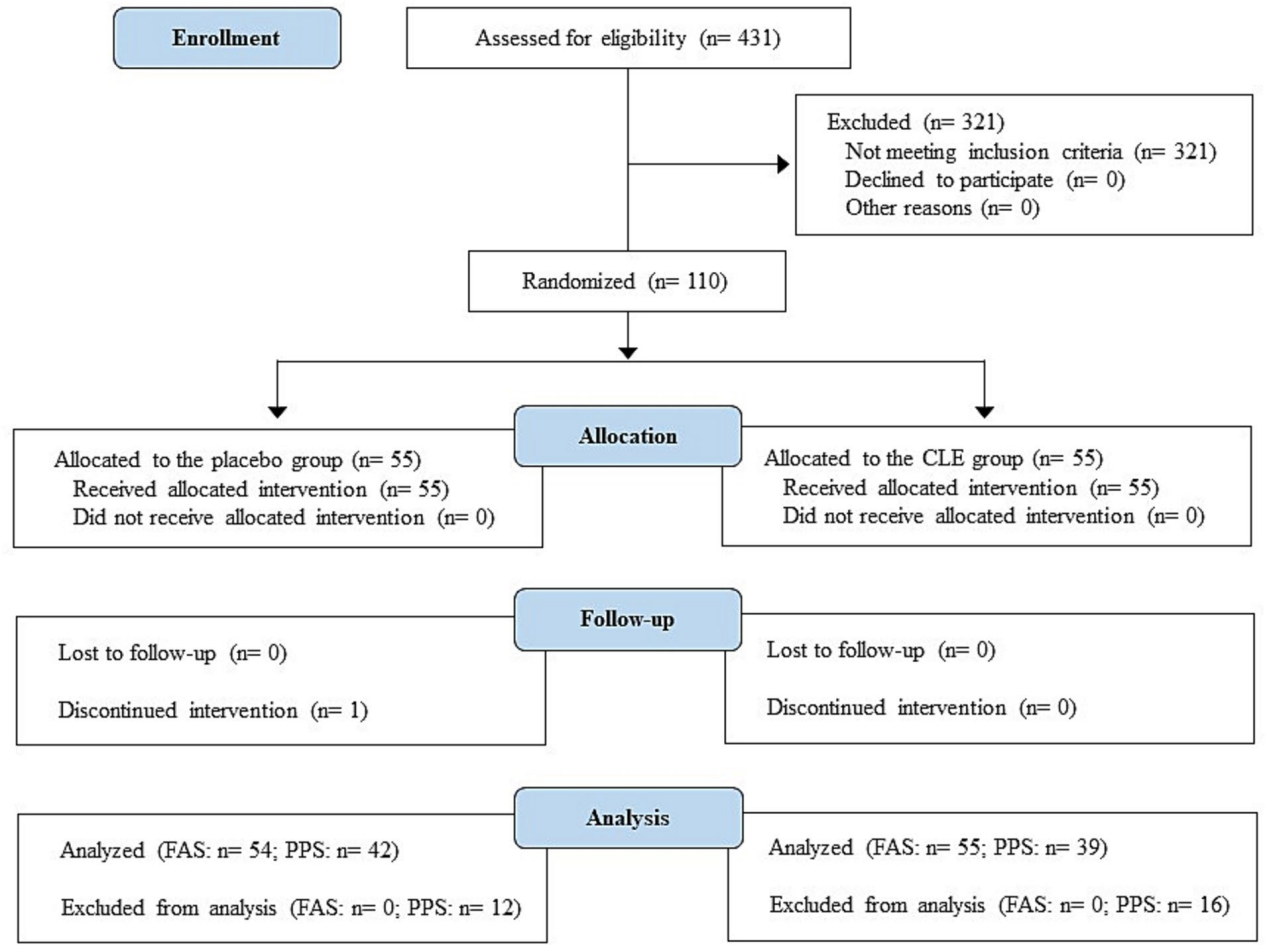
Study flow diagram (CONSORT 2010). CLE, a mixture of a hot water extract and a supercritical carbon dioxide extract of *Curcuma longa*; FAS, full analysis set; PPS, per-protocol set.

After the non-clinical study, we performed a 12-week, randomized, double-blind, placebo-controlled study from July to December 2021 at the Chiyoda Paramedical Care Clinic in Tokyo, Japan, by a contract research organization (CRO; CPCC Co., Ltd., Tokyo, Japan). The study was registered with the University hospital Medical Information Network (UMIN; registration number, UMIN000045106), approved by the institutional review board of Chiyoda Paramedical Care Clinic, and performed in accordance with the principles of the Declaration of Helsinki. All participants provided written informed consent prior to their enrollment in the study. The Consolidated Standards of Reporting Trials (CONSORT) 2010 flowchart and checklist (48) are provided as Figure 2 and Supplementary Table S1, respectively.

#### 2.2.2 Enrollment of participants

A total of 431 consecutive male and female patients at Chiyoda Paramedical Care Clinic were recruited and assessed for eligibility from July to September 2021. The inclusion criteria were age 40 to 69 years, overweight (body mass index [BMI] ≥ 23 to <30 kg/m^2^) (49), and normal/prediabetes glycemia (fasting plasma glucose, < 126 mg/dL; 2-h post-load plasma glucose during OGTT, < 200 mg/dL) (50). The exclusion criteria were (1) current or planned dietary carbohydrate restriction with the aim to lose weight during the scheduled study period; (2) continued intake 3 or more times per week of medications or nutraceutical/functional foods that may influence the results of this study; (3) planned change to daily diet and/or exercise habits and living environment (e.g., moving house or relocating for work) during the planned study period; (4) extremely irregular meal timings; (5) excessive alcohol intake (mean daily consumption ≥60 g); (6) smoking (mean daily consumption ≥ two packs); (7) positive hepatitis C virus antibody or hepatitis B surface antigen test; (8) drug or food allergies (in particular soybeans and gelatin); (9) recent (past 4 weeks), current, or planned participation in another clinical study during the study period; (10) pregnancy or breastfeeding in women or planned pregnancy during the study period; (11) history of or current treatment for a severe disease; (12) blood donation of 200 mL in the past month; (13) if male, blood donation of 400 mL in the past 3 months; (14) if female, blood donation of 400 mL in the past 4 months; (15) if male, total blood donation of more than 1,200 mL, calculated as the sum of donations in the past year and the estimated sampling amount in the study; (16) if female, total blood donation of more than 800 mL, calculated as the sum of donations in the past year and the estimated sampling amount in the study; and (17) deemed unsuitable for the study by the study investigators.

Of the 431 screened participants, 110 were found to be suitable for inclusion and enrolled. Participants were randomly allocated to the study groups by a stratified randomization method that considered sex, age, BMI, fasting and postprandial glucose, and high-sensitivity CRP (hsCRP) level (CLE group, *n* = 55; placebo group, *n* = 55). The authors randomly assigned numbers to the CLE and placebo capsules, and the CRO did the same for the participants; the randomization lists were kept in a secure location until locking of the database. All participants and study investigators were blinded to the group allocation.

#### 2.2.3 Experimental foods

Table 1 details the test capsule composition. CLE and placebo base capsules were composed of gelatin, glycerin, soybean-derived emulsifier, and beeswax. The CLE was the same as that tested in the RAW264.7 macrophages (see Section 2.1). The coloring agents carob and tartrazine were added to placebo capsules to give them the same appearance as CLE capsules.

**Table 1:** Composition of the test capsules.

	Placebo (0.97 g/2 capsules)	CLE (0.97 g/2 capsules)
Energy, Kcal	6.6	5.6
Carbohydrate, g	0.10	0.25
Protein, g	0.23	0.24
Lipid, g	0.59	0.40
Sodium chloride, mg	1.7	1.9
Bisacurone, μg	0	400
Turmeronol A, μg	0	100
Turmeronol B, μg	0	100

CLE, a mixture of a hot water extract and a supercritical carbon dioxide extract of *Curcuma longa*.

#### 2.2.4 Intervention

The intervention period was from September to December 2021. During the 12-week study, participants ingested two CLE or placebo capsules/day. Study visits were performed at 0, 4, 8, and 12 weeks and consisted of an interview with an experienced physician, physical assessments, and an OGTT. At the same visits, hematology and biochemistry tests and urinalysis were performed by a contracted laboratory company (BML, Inc., Tokyo, Japan). Before study visits, participants were allowed to consume only water from 9:00 pm on the evening before.

During the study, participants completed a daily diary with information on disease occurrence and symptoms; consumption of test capsules, nutraceutical/functional foods, and drugs (reasons for taking them, dose, and length of use); vaccinations (reasons for vaccination, date of vaccination, and adverse effects); exercise (exercise or no exercise, days of exercise, duration of exercise); any dietary carbohydrate restriction or dieting to lose weight (duration of dietary carbohydrate restriction/dieting); sleep duration (sleeping hours); and change of living environment. All participants were instructed to comply with the following study rules throughout the 12 weeks: (1) maintain usual lifestyle, including dietary, exercise, alcohol use, and smoking habits and use of medicine; (2) avoid consuming nutraceutical/functional foods (excluding study capsules); (3) do not donate blood; and (4) do not drink excessive amounts of alcohol or smoke a large number of cigarettes, do not perform extreme amounts of exercise, and do not fast or consume an unbalanced diet.

#### 2.2.5 Measurement of blood parameters

The hsCRP serum level and HbA1c were measured by standard latex agglutination turbidimetry, plasma glucose was measured by the hexokinase method, and serum insulin was measured with chemiluminescent immunoassay.

#### 2.2.6 The OGTT procedure

After a blood sample was obtained for measurement of fasting glucose and insulin levels, participants ingested a solution containing 75 g of glucose. Blood sampling was conducted after 30, 60, and 120 min for measurement of post-load blood glucose and insulin levels, and the areas under the curve (AUCs), times to peak concentration, insulinogenic indices, and insulin sensitivity indices were calculated.

#### 2.2.7 Calculation of insulinogenic index

The insulinogenic index was used to evaluate insulin secretory capacity in the first phase (within 30 min) after glucose load. It was calculated according to the following formula: insulinogenic index = [insulin _30 min_ – insulin _0 min_]/[glucose _30 min_ – glucose _0 min_]. A low score for this index indicates impaired insulin secretion capacity and is also used as a predictor of type 2 diabetes (51).

#### 2.2.8 Calculation of insulin sensitivity indices

Both the Cederholm index and the Stumvoll index, which are calculated from the OGTT values of glucose and insulin, were used as indices of estimated insulin sensitivity. These indices are highly correlated with the results of an insulin clamp test, which can accurately quantify insulin sensitivity but is not suitable for large-scale studies because it is laborious and expensive (52). These indices were calculated according to the following formula: Cederholm index = [75,000 + (glucose _0 min_ – glucose _120 min_) × 1.15 × 180 × 0.19 × body weight]/[(120 × mean glucose _0–120 min_) × log (mean insulin _0–120 min_)]; Stumvoll index = 0.156–0.0000459 × insulin _120 min_ – 0.000321 × insulin _0 min_ – 0.00541 × glucose _120 min_. Low scores for these indices indicate reduced insulin sensitivity and are reported to be associated with an increased risk of type 2 diabetes (53). Previous intervention studies showed that anti-diabetic drugs and functional foods improve not only the scores of the Cederholm and Stumvoll indices but also postprandial glucose levels (54, 55).

#### 2.2.9 Sample size

To calculate the minimum sample size necessary to ensure adequate statistical power, we used the G Power 3.1.9 program (University of Dusseldorf, Germany) and results of a previous clinical study. In that study, an aqueous extract of *Adansonia digitata* L., which is known to have anti-inflammatory activities (56), reduced the change in postprandial glucose levels from 0 min to 60 and 120 min after glucose load by at least 25% compared with the placebo group (57). The required sample size was calculated to be at least 39 participants per group based on the following assumption: 25% reduction in post-load glucose by CLE, Cohen’s d value of 0.65, statistical power of 80%, and type 1 error of 5%. Some previous clinical studies that used OGTT estimated the required sample size to be 50 to 55 participants per group, assuming a maximum dropout rate of 20% (58, 59). Therefore, in the present study, the sample size was determined to be 55 participants per group.

#### 2.2.10 Statistical analysis

Data from all randomized participants, i.e., the intention-to-treat (ITT) population, were included in the statistical analysis. Safety was evaluated in the full analysis set (FAS), and efficacy was analyzed in the per-protocol set (PPS); the PPS did not include participants who missed any study visits. The IBM SPSS statistical software package (version 26) for Windows (IBM Corp., Armonk, NY, USA) was used for all statistical analyses. Results are shown as mean (SD). Intergroup comparisons of baseline characteristics were performed with the two-tailed unpaired Student’s *t* test in case of homogeneous variance and the Aspin-Welch *t* test in case of heterogeneous variance, whereby sex and urinalysis were analyzed by a two-tailed Mann–Whitney *U* test. To analyze changes from baseline, we used repeated measures two-way ANOVA (two groups × three-time points) and an SPSS general linear model to determine the main effects of group, time, and group × time; then, we used simple main effect tests to compare groups at each visit (22, 60–62). The statistical significance level was set at 0.05.

## 3 Results

### 3.1 Non-clinical study

#### 3.1.1 Effects of CLE and its potential active components on the production of inflammatory mediators in RAW264.7 macrophage cells stimulated with LPS

LPS stimulation markedly increased the production of IL-1β, IL-6, and NO in LPS-stimulated RAW264.7 cells compared with unstimulated control cells, but the production of these substances was significantly inhibited by pretreatment of cells with CLE and turmeronol A, turmeronol B, and bisacurone at the concentrations contained in the CLE (Supplementary Figure S1). LPS stimulation also increased TNF-α protein production, but the increase was significantly inhibited by pretreatment of cells with CLE and all test compounds except turmeronol B at the concentrations contained in the CLE (Supplementary Figure S1).

### 3.2 Clinical intervention study

#### 3.2.1 Participants

The flowchart of participants in the study is shown in Figure 2. A total of 110 out of 431 potential participants were randomly allocated to the CLE or placebo group (*n* = 55 per group). One participant in the placebo group declined to continue for personal reasons. In addition, some participants (*n* = 12 in the placebo group and *n* = 16 in the CLE group) were excluded from the efficacy assessment (PPS analysis) because they did not comply with the protocol for the following reasons: use of health foods (*n* = 3 in the placebo group and *n* = 1 in the CLE group), medical treatment of chronic diseases (*n* = 1 in the placebo group [lumbar spondylosis] and *n* = 3 in the CLE group [hypertension, hyperuricemia, and adipoma]), change of lifestyle during the scheduled study period (*n* = 2 in the CLE group), irregular dietary habits (*n* = 1 in the placebo group), and unsuitable for the study for other reasons (unreliable study results because of hemolysis during blood collection [*n* = 1 in the CLE group], symptoms with acute inflammation [*n* = 1 in the placebo group due to a common cold and *n* = 1 in the CLE group due to injuries], excessive exercise before visiting the study center [*n* = 1 in the placebo group and *n* = 1 in the CLE group], and suspected adverse effects of the Covid-19 vaccine (63) [*n* = 5 in the placebo group and *n* = 7 in the CLE group]). The other 81 participants were included in the PPS analysis.

The baseline characteristics were not significantly different between the two groups, but the Cederholm index was significantly higher in the CLE group (Tables 2, 3). The mean intake of test capsules was also not significantly different between the two groups (data not shown). During the study period, some participants exercised (*n* = 22 in the placebo group and *n* = 17 in the CLE group) and some did not (*n* = 20 in the placebo group and *n* = 22 in the CLE group); the number of participants who exercised did not differ significantly between the two groups (*p* = 0.507). In addition, the mean (SD) number of days of exercise was not significantly different between the two groups (placebo group, 11.0 [17.6] days; CLE group, 9.3 [17.7] days; *p* = 0.666).

**Table 2:** Baseline characteristics of the participants^1^.

	Placebo group (*n* = 42)		CLE group (*n* = 39)		*p* value
	Mean	SD	Mean	SD	
Sex, male/female, *n*	19/23		24/15		0.539
Age, years	54.7	6.5	55.1	7.3	0.815
Physical measurements and tests					
Height, cm	163.5	8.0	164.5	7.0	0.571
Body weight, kg	69.8	7.6	70.7	6.7	0.555
BMI, kg/m^2^	26.0	1.3	26.1	1.4	0.821
SBP, mmHg	127.3	13.3	125.3	13.5	0.509
DBP, mmHg	81.4	9.9	80.6	10.4	0.743
Serum inflammatory markers					
hsCRP, mg/dL	0.116	0.118	0.105	0.090	0.656
Metabolic markers					
Glucose, mg/dL	94.2	7.3	95.0	7.8	0.630
Insulin, μU/mL	6.2	2.6	5.3	2.6	0.121
HbA1c, %	5.46	0.30	5.47	0.28	0.852

BMI, body mass index; CLE, a mixture of a hot water extract and a supercritical carbon dioxide extract of *Curcuma longa*; DBP, diastolic blood pressure; HbA1c, hemoglobin A1c; hsCRP, high-sensitivity C-reactive protein; SBP: systolic blood pressure. ^1^Values represent the means and standard deviations. The sex distribution in each group was compared with the two-tailed Mann–Whitney U test. Physical measurements and tests, serum inflammatory markers, and metabolic markers were compared with the two-tailed unpaired Student’s *t* test when variance was homogeneous or the Aspin-Welch *t* test when variance was heterogeneous.

**Table 3:** Baseline values for post-load glucose and insulin, insulinogenic index, and insulin sensitivity indices in the oral glucose tolerance test (OGTT)^1^.

	Placebo group (*n* = 42)		CLE group (*n* = 39)		*p* value
	Mean	SD	Mean	SD	
Postprandial glucose					
0 min, mg/dL	94.2	7.3	95.0	7.8	0.630
30 min, mg/dL	155.7	23.4	158.1	22.4	0.645
60 min, mg/dL	183.6	29.2	175.5	30.9	0.228
120 min, mg/dL	152.6	16.8	149.5	22.3	0.480
Early phase AUC_0–60 min_, mg·hr./dL	147.3	16.6	146.6	16.8	0.863
Late phase AUC_60–120 min_, mg·hr./dL	168.1	14.9	162.5	18.5	0.136
Total AUC_0–120 min_, mg·hr./dL	315.4	28.6	309.2	32.5	0.360
Time to peak concentration, min	62.1	21.4	64.6	26.2	0.642
Postprandial insulin					
0 min, μU/mL	6.2	2.6	5.3	2.6	0.121
30 min, μU/mL	43.0	30.0	39.9	28.4	0.633
60 min, μU/mL	51.1	24.7	49.5	31.5	0.791
120 min, μU/mL	75.3	33.2	66.5	38.5	0.273
Early phase AUC_0–60 min_, μU/mL	35.8	19.1	33.6	20.4	0.616
Late phase AUC_60–120 min_, μU/mL	63.2	24.9	58.0	33.2	0.423
Total AUC_0–120 min_, μU/mL	99.0	40.4	91.6	51.0	0.467
Time to peak concentration, min	101.4	32.4	103.1	29.8	0.813
Insulinogenic index	0.58	0.35	0.57	0.41	0.824
Insulin sensitivity indices					
Cederholm index	39.4	6.0	43.0	9.3	0.044
Stumvoll index	0.0723	0.0143	0.0780	0.0185	0.118

AUC, area under the curve; CLE: a mixture of a hot water extract and a supercritical carbon dioxide extract of *Curcuma longa*. ^1^Values represent the means and standard deviations. Post-load glucose and insulin and estimated insulin sensitivities were compared with the two-tailed unpaired Student’s *t* test when variance was homogeneous or the Aspin-Welch *t* test when variance was heterogeneous.

#### 3.2.2 Effect of CLE on body weight and BMI

During the entire study period, the change in body weight from week 0 showed no significant differences between the two groups (repeated measures two-way ANOVA [r-ANOVA]), but in week 4, it was significantly higher in the CLE group than in the placebo group. The change in BMI from week 0 was also not significantly different between the two groups throughout the whole study period (r-ANOVA) but was significantly higher in the CLE group than in the placebo group in week 4. The change in body weight and BMI from week 0 showed no significant differences between the two groups at weeks 8 and 12 (data not shown).

#### 3.2.3 Effect of CLE on serum hsCRP level

The change in hsCRP from week 0 showed no significant differences between the two groups throughout the whole study period (r-ANOVA), but it was significantly lower in the CLE group than in the placebo group at weeks 4 and 12 (Table 4).

**Table 4:** Effect of *Curcuma longa* extract (CLE) on the change of fasting blood markers of systemic inflammation and glucose metabolism^1^.

	Change from baseline (Week 0)						Repeated measures two-way ANOVA		
	Week 4		Week 8		Week 12		Group	Time	Interaction
	Mean	SD	Mean	SD	Mean	SD			
hsCRP, mg/dL									
Placebo	0.016	0.132	−0.012	0.105	0.006	0.120	0.130	0.817	0.070
CLE	−0.037**	0.080	−0.012	0.130	−0.034**	0.079			
Glucose, mg/dL									
Placebo	3.26	8.39	3.10	6.18	2.79	7.69	0.523	0.626	0.941
CLE	2.74	5.32	2.53	5.89	1.82	6.55			
Insulin, μU/mL									
Placebo	1.59	2.86	0.16	3.13	0.77	2.89	0.957	0.088	0.531
CLE	1.00	1.95	0.65	1.79	0.48	2.25			
HbA1c, %									
Placebo	−0.03	0.13	0.00	0.14	0.00	0.17	0.184	0.528	0.077
CLE	−0.04	0.17	−0.04	0.18	−0.08**	0.19			

ANOVA, analysis of variance; CLE: a mixture of a hot water extract and a supercritical carbon dioxide extract of *Curcuma longa*; HbA1c: hemoglobin A1c; hsCRP: high-sensitive C-reactive protein. ^1^Values represent the means and standard deviations at 4, 8, and 12 weeks for *n* = 42, 40, and 42 (placebo group) or *n* = 39, 38, and 39 (CLE group), respectively. Significant differences were evaluated by repeated measures two-way ANOVA, followed by a simple main effect test (**p < 0.01 compared with the placebo group at each indicated time point).

#### 3.2.4 Effect of CLE on fasting glucose, insulin, and HbA1c levels

The changes in fasting plasma glucose and serum insulin from week 0 were not significantly different between the two groups (Table 4). Although the change in HbA1c from week 0 showed no significant differences between the two groups throughout the whole study period (r-ANOVA), at week 12, it was significantly lower in the CLE group than in the placebo group (Table 4).

#### 3.2.5 Effect of CLE on post-load glucose and insulin in OGTT

##### 3.2.5.1 Week 0

The changes in glucose and insulin from 0 min were not significantly different between the two groups (Table 5). The change in insulin from 0 min showed no significant differences between the two groups throughout the OGTT (r-ANOVA), but at 120 min it tended to be lower in the CLE group than in the placebo group (*p* = 0.093) (Table 5).

**Table 5:** Effect of *Curcuma longa* extract (CLE) on the change of post-load glucose and insulin levels during the oral glucose tolerance test (OGTT)^1^.

	Change from baseline (0 min)						Repeated measures two-way ANOVA		
	30 min		60 min		120 min		Group	Time	Interaction
	Mean	SD	Mean	SD	Mean	SD			
Week 0									
Glucose, mg/dL									
Placebo	61.5	20.2	89.4	27.1	58.5	18.4	0.233	< 0.001	0.388
CLE	63.1	21.4	80.5	29.8	54.6	22.5			
Insulin, μU/mL									
Placebo	36.8	28.7	44.9	23.9	69.0	32.6	0.550	< 0.001	0.467
CLE	34.6	26.8	44.1	29.8	61.1	36.9			
Week 4									
Glucose, mg/dL									
Placebo	56.4	13.8	67.4	34.3	40.6	28.2	0.380	< 0.001	0.228
CLE	59.5	20.1	62.9	33.9	31.3	35.1			
Insulin, μU/mL									
Placebo	45.1	25.9	45.8	23.8	66.5	36.7	0.498	0.009	0.290
CLE	47.0	38.2	47.6	33.5	54.2	39.5			
Week 8									
Glucose, mg/dL									
Placebo	59.6	17.9	73.2	29.6	32.1	29.8	0.459	< 0.001	0.506
CLE	59.7	20.3	65.2	31.4	27.1	25.6			
Insulin, μU/mL									
Placebo	46.9	35.3	57.2	27.9	62.6	38.1	0.550	0.002	0.963
CLE	42.6	21.4	53.0	38.9	56.1	36.5			
Week 12									
Glucose, mg/dL									
Placebo	56.4	19.2	70.9	33.8	31.0	26.2	0.045	< 0.001	0.029
CLE	59.5	20.4	56.2**	37.2	16.7**	22.5			
Insulin, μU/mL									
Placebo	39.9	21.3	48.9	26.6	62.3	34.3	0.193	0.006	0.005
CLE	43.7	28.3	45.8	25.9	43.7**	24.0			

ANOVA, analysis of variance; CLE: a mixture of a hot water extract and a supercritical carbon dioxide extract of *Curcuma longa*. ^1^Values represent the means and standard deviations at 4, 8, and 12 weeks for *n* = 42, 40, and 42 (placebo group) or *n* = 39, 38, and 39 (CLE group), respectively. Significant differences were evaluated by repeated measures two-way ANOVA, followed by a simple main effect test (**p < 0.01 compared with the placebo group at each indicated time point).

##### 3.2.5.2 Week 4

At week 4, the change in glucose from 0 min showed no significant differences between the two groups throughout the OGTT (r-ANOVA), but it tended to be lower in the CLE group than in the placebo group at 120 min (*p* = 0.078) (Table 5). The change in insulin from 0 min was not significantly different between the two groups throughout the OGTT (r-ANOVA), but it tended to be lower in the CLE group than in the placebo group at 120 min (*p* = 0.084) (Table 5).

##### 3.2.5.3 Week 8

At week 8, the changes in glucose and insulin from 0 min showed no significant differences between the two groups (Table 5).

##### 3.2.5.4 Week 12

**Figure 3:**
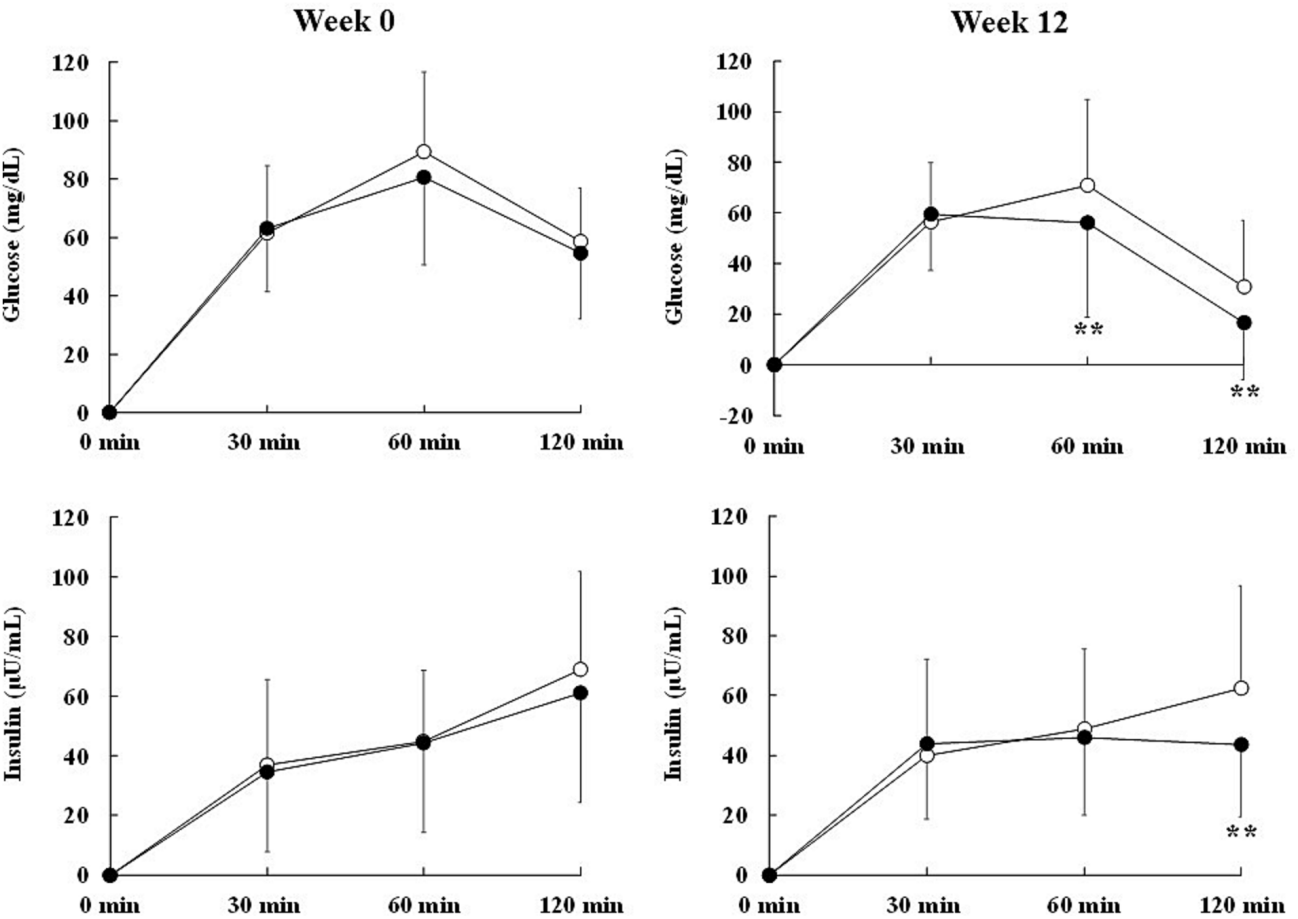
Effect of *Curcuma longa* extract (CLE) on the change in marker levels of post-load glycometabolism during the oral glucose tolerance test (OGTT) in middle-aged and elderly participants with overweight and glycemia in the normal/prediabetes range. Glucose and insulin levels after oral glucose load in the OGTT were measured at baseline and after 12 weeks of consuming test capsules containing CLE (CLE group; black circle, *n* = 39) or placebo (placebo group; white circle, *n* = 42). Data are means and standard deviations. Mean values in the CLE group were significantly different from those in the placebo group. **p < 0.01 (repeated measures two-way analysis of variance, followed by a simple main effect test). CLE, a mixture of a hot water extract and a supercritical carbon dioxide extract of *Curcuma longa*; OGTT, oral glucose tolerance test.

At week 12, the change in glucose from 0 min was significantly lower in the CLE group than in the placebo group throughout the OGTT (r-ANOVA) and at 60 min and 120 min (Table 5; Figure 3). The change in insulin from 0 min showed no significant differences between the two groups throughout the OGTT (r-ANOVA), but it was significantly lower in the CLE group than in the placebo group at 120 min (Table 5; Figure 3).

#### 3.2.6 Effect of CLE on post-load values at 120 min and AUC of glucose and insulin during the OGTT

The change from week 0 in post-load glucose at 120 min showed no significant differences between the two groups throughout the OGTT (r-ANOVA), but it was significantly lower in the CLE group than in the placebo group at week 12 (Table 6). The change from week 0 in post-load insulin at 120 min showed no significant differences between the two groups throughout the OGTT (r-ANOVA), but it tended to be lower in the CLE group than in the placebo group at week 12 (*p* = 0.097) (Table 6). The changes from week 0 in the AUC of early-phase glucose and insulin in the first hour after administration of oral glucose (AUC_0–60 min_) showed no significant differences between the two groups (Table 6). The change from week 0 in the AUC of late-phase glucose (AUC_60–120 min_) was not significantly different between the two groups throughout the OGTT (r-ANOVA) but was significantly lower in the CLE group than in the placebo group at week 12 (Table 6), and the change from week 0 in the AUC of late-phase insulin (AUC_60–120 min_) showed no significant differences between the two groups throughout the OGTT (r-ANOVA) but tended to be lower in the CLE group than in the placebo group at week 12 (*p* = 0.066) (Table 6). The change from week 0 in the AUC of total glucose (AUC_0–120 min_) showed no significant differences between the two groups throughout the OGTT (r-ANOVA) but tended to be lower in the CLE group than in the placebo group at week 12 (*p* = 0.079) (Table 6), and the change from week 0 in the AUC of total insulin (AUC_0–120 min_) showed no significant differences between the two groups (Table 6).

**Table 6:** Effect of *Curcuma longa* extract (CLE) on the change in 120-min post-load glucose and insulin values, area under the curve (AUC), and time to peak concentrations of glucose and insulin in the oral glucose tolerance test (OGTT)^1^.

	Change from baseline (Week 0)						Repeated measures two-way ANOVA		
	Week 4		Week 8		Week 12		Group	Time	Interaction
	Mean	SD	Mean	SD	Mean	SD			
120-min postprandial values									
Glucose, mg/dL									
Placebo	−14.6	30.2	−24.6	32.3	−24.7	26.0	0.231	0.001	0.406
CLE	−20.5	35.6	−25.7	26.3	−36.1*	26.7			
Insulin, μU/mL									
Placebo	−0.9	35.0	−7.9	39.4	−5.9	33.0	0.228	0.044	0.668
CLE	−6.0	34.4	−5.1	23.7	−17.0	32.1			
Early phase AUC_0–60 min_									
Glucose, mg·hr./dL									
Placebo	−4.8	16.9	−2.2	15.6	−4.4	15.6	0.856	0.616	0.675
CLE	−3.4	17.8	−4.0	13.4	−6.1	19.2			
Insulin, μU·hr./mL									
Placebo	6.0	20.4	8.2	16.8	3.4	19.0	0.811	0.208	0.493
CLE	8.1	17.9	6.6	13.1	5.5	14.3			
Late phase AUC_60–120 min_									
Glucose, mg·hr./dL									
Placebo	−16.7	21.4	−19.2	23.1	−20.2	20.0	0.347	0.024	0.246
CLE	−17.7	30.2	−19.8	17.1	−29.3*	21.3			
Insulin, μU·hr. /mL									
Placebo	0.8	19.9	2.5	26.9	−0.6	23.4	0.437	0.0497	0.425
CLE	−0.8	20.3	2.2	16.6	−7.4	20.7			
Total AUC_0–120 min_									
Glucose, mg·hr. /dL									
Placebo	−21.5	33.6	−21.5	33.2	−24.6	31.2	0.479	0.105	0.403
CLE	−21.1	44.3	−23.8	26.1	−35.4	36.4			
Insulin, μU·hr. /mL									
Placebo	6.8	30.9	10.7	38.7	2.8	37.1	0.706	0.032	0.783
CLE	7.3	30.5	8.8	24.7	−1.9	27.5			
Time to peak concentration									
Glucose, min									
Placebo	−4.3	30.8	−6.0	29.8	−5.7	24.1	0.184	0.932	0.871
CLE	−13.8*	36.3	−12.6	36.7	−14.6*	32.9			
Insulin, min									
Placebo	−11.4	48.7	−26.3	40.3	−7.1	44.8	0.178	0.372	0.011
CLE	−21.5	45.1	−22.9	47.5	−33.8**	41.4			

ANOVA, analysis of variance; AUC, area under the curve; CLE, a mixture of a hot water extract and a supercritical carbon dioxide extract of *Curcuma longa*. ^1^Values represent the means and standard deviations at 4, 8, and 12 weeks for *n* = 42, 40, and 42 (placebo group) or *n* = 39, 38, and 39 (CLE group), respectively. Significant differences were evaluated by repeated measures two-way ANOVA, followed by a simple main effect test (*p < 0.05 and **p < 0.01 compared with the placebo group at each indicated time point).

#### 3.2.7 Effect of CLE on time to peak concentration of glucose and insulin during the OGTT

The change from week 0 in the time to peak concentration of glucose showed no significant differences between the two groups throughout the whole study period (r-ANOVA) but was significantly lower in the CLE group than in the placebo group at weeks 4 and 12 (Table 6). The change from week 0 in the time to peak concentration of insulin showed no significant differences between the two groups throughout the whole study period (r-ANOVA) but was significantly lower in the CLE group than in the placebo group at week 12 (Table 6).

#### 3.2.8 Effect of CLE on insulinogenic index

The changes from week 0 in the insulinogenic index were not significantly different between the two groups (Table 7).

**Table 7:** Effect of *Curcuma longa* extract (CLE) on the change of insulinogenic indexes and insulin sensitivity indices^1^.

	Change from baseline (Week 0)						Repeated measures two-way ANOVA		
	Week 4		Week 8		Week 12		Group	Time	Interaction
	Mean	SD	Mean	SD	Mean	SD			
Insulinogenic index									
Placebo	0.28	0.65	0.28	0.54	0.28	0.83	0.463	0.716	0.662
CLE	0.51	1.64	0.23	0.34	0.29	0.72			
Insulin sensitivity indices									
Cederholm index									
Placebo	3.5	7.2	4.5	7.6	5.9	8.3	0.363	0.003	0.440
CLE	4.2	10.4	4.9	7.5	8.8*	10.3			
Stumvoll index									
Placebo	−0.0033	0.0134	0.0096	0.0216	0.0076	0.0170	0.261	< 0.001	0.098
CLE	−0.0003	0.0109	0.0079	0.0146	0.0152**	0.0168			

ANOVA, analysis of variance; CLE, a mixture of a hot water extract and a supercritical carbon dioxide extract of *Curcuma longa*. ^1^Values represent the means and standard deviations at 4, 8, and 12 weeks for *n* = 42, 40, and 42 (placebo group) or *n* = 39, 38, and 39 (CLE group), respectively. Significant differences were evaluated by repeated measures two-way ANOVA, followed by a simple main effect test (*p < 0.05 and **p < 0.01 compared with the placebo group at each indicated time point).

#### 3.2.9 Effect of CLE on insulin sensitivity indices

The Cederholm index showed no significant differences in the change from week 0 between the two groups throughout the whole study period (r-ANOVA), but the change was significantly higher in the CLE group than in the placebo group at week 12 (Table 7). Similarly, the Stumvoll index showed no significant differences in the change from week 0 between the two groups throughout the whole study period (r-ANOVA), but the change was significantly higher in the CLE group than in the placebo group at week 12 (Table 7).

#### 3.2.10 Safety of the intervention

Adverse events were assessed in the ITT population (placebo group, *n* = 55; CLE group, *n* = 55). In the placebo group, the following adverse events occurred: headache and shoulder pain (one case), toothache (one case), pharyngeal pain (two cases), arm pain (one case), abdomen pruritus (one case), stomach upset and diarrhea (one case), colon polyp (one case), common cold symptoms (seven cases), rhinitis (one case), diarrhea (one case), insect bite (one case), lumbar spondylosis (one case), increased uric acid (one case), positive urinary protein (four cases), positive urinary blood (one case), increased triglyceride (two cases), increased creatine phosphokinase (three cases), increased aspartate aminotransferase (one case), and increased body weight (five cases). In the CLE group, the following adverse events occurred: headache (three cases), eye pain (one case), bloodshot eyes (one case), infective conjunctivitis (one case), tooth decay (one case), bleeding and swelling of the gums (one case), pharyngeal pain (one case), shoulder adipoma (one case), fever and shoulder pain (two cases), fever and arm pain (one case), arm pain (one case), elbow bruise (one case), low back pain (one case), fatigue (one case), common cold symptoms (nine cases), diarrhea (one case), heat rash (one case), verruca vulgaris (one case), *helicobacter pylori* infection (one case), high blood pressure (one case), positive urinary glucose (one case), increased uric acid (one case), increased triglyceride (one case), increased creatine phosphokinase (three cases), increased CRP (two cases), increased alanine transaminase (one case), increased body weight (two cases), and decreased body weight (two cases). These adverse events were mild, and an experienced physician judged that they were unrelated to the dietary intervention.

Safety parameters (hematology and biochemistry tests, urinalysis, and physical measurements and tests) were assessed in the FAS population (placebo group, *n* = 54; CLE group, *n* = 55). The urinalysis and physical measurements were not significantly different between the two groups. The number of participants with reduced kidney function, defined as an estimated glomerular filtration rate (eGFR) below 60 mL/min/1.73 m^2^, was not significantly different between the CLE group (*n* = 5) and placebo group (*n* = 5) at week 0 but was significantly lower in the CLE group (*n* = 3) than in the placebo group (*n* = 10) at week 12 (chi-squared tests). The number of red blood cells was significantly lower in the CLE group than in the placebo group at weeks 4 and 8; the level of uric acid was significantly lower in the CLE group than in the placebo in week 8; and the level of total bilirubin at weeks 4 and 8 and chloride at week 4 was significantly higher in CLE group than in the placebo group. However, these changes were within the corresponding reference ranges.

## 4 Discussion

We investigated the effect of a mixture of a hot water extract and a supercritical carbon dioxide extract of *C. longa* (CLE) and its potential active ingredients (turmeronols A and B and bisacurone) on the production of inflammatory mediators in RAW264.7 macrophage cells stimulated with LPS. In addition, we performed a 12-week, randomized, double-blind, placebo-controlled study to investigate the effect of CLE on chronic inflammation and postprandial hyperglycemia in middle-aged and elderly participants with overweight and glycemia in the normal/prediabetes range. In the *in vitro* study, CLE, turmeronol A, and bisacurone significantly inhibited the production of IL-1β, IL-6, TNF-α, and NO in RAW264.7 cells stimulated with LPS, and turmeronol B significantly inhibited LPS-induced production of IL-1β, IL-6, and NO. In the clinical study, we found that intake of CLE significantly reduced serum hsCRP and HbA1c levels. Furthermore, in the OGTT, post-load plasma glucose level, time to peak concentration of glucose and insulin, and insulin sensitivity indices were significantly improved in the CLE group. These results suggest that CLE may improve insulin sensitivity and postprandial hyperglycemia by reducing chronic inflammation and that this reduction is due at least partly to the anti-inflammatory effects of turmeronols A and B and bisacurone.

Chronic inflammation is induced by several triggers, such as aging, accumulation of visceral fat, and unhealthy lifestyle. It is sustained by inflammatory mediators produced by inflammatory cells such as macrophages and is involved in the development of metabolic abnormalities and various inflammatory diseases (1). In the present study, CLE and its potential active compounds, i.e., turmeronols A and B and bisacurone, inhibited the production of inflammatory mediators in activated macrophages stimulated with LPS (Supplementary Figure S1). Considering that 600 μg/mL CLE contains 1.1 μM turmeronol A, 1.1 μM turmeronol B, and 4.2 μM bisacurone and that the sum of anti-inflammatory activity of the three potential active compounds appears to be comparable to that of CLE, all the anti-inflammatory effects of CLE may be attributable to the turmeronols and bisacurone. In previous animal studies, a hot water extract of *C. longa* containing turmeronols A and B and bisacurone (22) not only inhibited the mRNA expression of inflammatory mediators, including IL-1β, IL-6, TNF-α, and inducible NO synthase (iNOS), but also ameliorated various inflammatory pathologies in mouse models, such as ethanol-induced liver injury and non-alcoholic steatohepatitis (36). As mentioned above, CRP is generally used as a systemic inflammatory marker in human clinical studies. Recently, it was reported that the hsCRP assay, which can detect small increases in CRP levels, can be used as a marker of low-grade inflammation (5, 7). Epidemiological studies have reported that slightly higher hsCRP (approximately 0.1 mg/dL) is associated with an increased risk of various inflammatory diseases, including metabolic syndrome (8), diabetes (9), coronary heart disease (11), and cancer (12). The risk of these diseases may be decreased by anti-inflammatory drugs (64–66). In a previous intervention study, *C. longa* extracts containing turmeronols and bisacurone were shown to decrease the level of systemic inflammatory markers, such as hsCRP, TNF-α, IL-6, and soluble vascular cell adhesion molecule-1, and to improve health-related quality of life (22, 23). In the present study, we also confirmed that CLE significantly reduces the serum level of hsCRP (Table 4). These results suggest that CLE, which contains turmeronols A and B and bisacurone, may reduce chronic low-grade inflammation and thus may have the potential to reduce the risk of chronic inflammatory diseases.

Generally, systemic glucose tolerance is evaluated with an OGTT. The 2-h OGTT glucose value is needed for diagnosing the presence of prediabetes and diabetes, and an elevated 2-h post-load glucose is known to be associated with an increased risk of diabetes (50). Individuals with prediabetes were reported to exhibit increased plasma levels of glucose during the OGTT before showing increases in fasting blood glucose levels (67). A higher postprandial glucose level is an early sign of prediabetes and is associated with an increased risk of type 2 diabetes (15, 68). Impaired postprandial glucose metabolism is associated with chronic inflammation (69). For example, the administration of clodronate liposome, an agent that selectively depletes systemic macrophages, was shown to reduce systemic inflammation and improve postprandial glucose levels in dietary-induced obese mouse models (70, 71). Human studies reported that hsCRP levels were more strongly positively correlated with postprandial than with fasting blood glucose levels (69). Furthermore, a clinical intervention study showed that salsalate, an anti-inflammatory drug, improved hsCRP and postprandial glucose levels (72). In addition, large-scale observation studies found that people taking anti-inflammatory drugs such as aspirin have a lower risk of type 2 diabetes than people not taking such drugs (73). In the present study, CLE not only significantly inhibited the elevation of post-load glucose levels immediately after oral glucose load but also improved the level of post-load glucose 2 h later (Tables 5, 6). The CLE group also had a significantly shorter time to peak glucose concentration than the placebo group (Table 6). Previous clinical studies found that after glucose ingestion, insulin levels decreased once blood glucose had returned to baseline (51, 74, 75). In line with these studies, in the present study, we also observed that CLE significantly decreased the level of post-load insulin at 120 min after a reduction in glucose level at 60 min (Table 5). Taken together, these findings suggest that CLE may reduce the postprandial elevation of glucose level and promote postprandial glucose clearance and, consequently, may be able to decrease the risk of prediabetes and type 2 diabetes.

The regulation of postprandial glycometabolism is involved in various processes, such as intestinal glucose absorption, insulin secretion by the pancreas, and insulin resistance (14, 76). In the gastrointestinal tract, dietary carbohydrates are digested by enzymes such as α-glucosidase into monosaccharides, which are absorbed in the small intestine (76). Acarbose, an α-glucosidase inhibitor that suppresses the intestinal absorption of glucose, is known to inhibit the elevation of postprandial glucose in the early phase after oral carbohydrate load (within 30 min) (77, 78), and a clinical study found that the reduction in the insulin secretory response to oral glucose load increases postprandial glucose levels (74). An insulinogenic index can be used as a marker of early-phase insulin secretory capacity associated with β-cell function (51), and the present study found no significant differences between the placebo and CLE groups in post-load glucose levels in the early phase and in the insulinogenic index (Tables 5, 7). Therefore, CLE may not have the potential to inhibit the intestinal absorption of glucose or to enhance insulin secretion by the pancreas.

Insulin resistance, also known as impaired insulin sensitivity, refers to impaired effects of insulin, such as reduced glucose uptake in insulin-sensitive tissues, including skeletal muscles, adipose tissues, and liver (14). In the fasting state, glucose uptake occurs mainly in non–insulin-sensitive tissues such as the brain, whereas in the postprandial state, approximately 80% of total body glucose uptake occurs in skeletal muscles (14, 79). Thus, skeletal muscle is the main tissue responsible for postprandial glucose uptake and glucose disposal (14, 80). However, the expression levels of phosphorylated proteins associated with insulin-mediated glucose uptake are lower in skeletal muscle tissues from obese participants than in those from lean participants (81). In fact, studies found that the rate of glucose uptake is 50% lower in the skeletal muscle of obese participants with impaired glucose tolerance (14, 82). In addition, previous clinical studies showed that in the OGTT, participants with impaired glucose tolerance are characterized by a significant increase in post-load glucose levels in the late phase (60 to 120 min) but not by a reduction of insulin secretion in the early phase (75, 83). In another clinical study, a stratified analysis showed that the level of postprandial glucose was significantly higher in overweight participants with a high CRP level than in those with a low CRP level but that it had no effect on the insulin secretory response after oral glucose load (17). Intervention studies found that in the OGTT, the level of late-phase glucose (AUC_60–120 min_) was improved by food ingredients such as 5-aminolevulinic acid that inhibit production of inflammatory mediators in RAW264.7 macrophage cells stimulated with LPS (84, 85). In addition, anti-inflammatory agents such as salsalate and rosiglitazone significantly improved insulin sensitivity and postprandial glucose levels in patients with obesity or diabetes, whereas the insulin response after oral glucose loading was not significantly different between the agent group and the placebo group (72, 86). Treatment with a water extract of *C. longa* increased glucose uptake in mouse skeletal muscle tissues under hyperglycemic culture conditions (87), and in the present study, CLE significantly improved post-load glucose levels and glucose AUC in the late phase (60 to 120 min) in the OGTT. Although no significant differences between the two groups were observed in early insulin response after oral glucose load, the Cederholm and Stumvoll insulin sensitivity indices both significantly improved in the CLE group (Table 7). Recently, insulin sensitivity was reported to influence the shape of the post-load glucose and insulin curves in the OGTT; impaired insulin sensitivity slows the onset of insulin action, such as glucose uptake, and causes a delay in the time until peak glucose and insulin concentrations in the OGTT (74, 88). In the present study, during the OGTT, the time to peak concentrations of glucose and insulin were both significantly shorter in the CLE group than in the placebo group (Table 6). These results suggest that CLE could potentially increase postprandial glucose uptake in skeletal muscles by improving impaired insulin sensitivity associated with chronic inflammation.

HbA1c represents the percentage of circulating glycated hemoglobin and is an indicator of average blood glucose levels over the past one to three months (67, 89). The HbA1c value is known to reflect not only elevated blood levels of fasting glucose but also hyperglycemia after daily meals such as breakfast, lunch, and dinner (90). Additionally, a clinical study found that in participants with an HbA1c level below 6.2%, approximately 70 to 90% of the total HbA1c value is attributable to postprandial hyperglycemia (89, 90). In an intervention study, anti-inflammatory drugs significantly reduced HbA1c and postprandial glucose levels (86, 91). Similar to these reports, in the present study, CLE also significantly improved post-load glucose and HbA1c levels (Tables 4, 5). These results suggest that CLE may help to decrease the blood level of HbA1c by suppressing the elevation of glucose levels after a meal.

Insulin, a polypeptide hormone that binds to the insulin receptor, causes phosphorylation of the insulin receptor substrate-1 (IRS-1) at tyrosine residues, activates phosphoinositide 3-kinase (PI3K)/protein kinase B (Akt) signaling pathways, and promotes the translocation of glucose transporter-4 from the cytoplasm into the cell membrane, where it induces glucose uptake into cells (92). The development of insulin resistance is caused by inflammation associated with macrophages, which are activated by recognition of factors such as free fatty acids and damage-associated molecular patterns (1, 5). Activated macrophages abundantly produce inflammatory mediators, which inhibit the insulin signaling pathway (6). As mentioned above, in obese mouse models, selectively eliminating macrophages by administering clodronate liposome reduced macrophage-induced systemic inflammation and consequently improved not only postprandial glucose levels but also insulin resistance (70, 71). Other studies showed that treatment with IL-1β or TNF-α inhibits insulin-induced tyrosine phosphorylation of IRS-1; increases serine/threonine phosphorylation of IRS-1, which can block insulin signaling; and impairs glucose uptake in response to insulin in various cells, such as skeletal muscle cells (93–96). A genetic deficiency of IL-1 or TNF receptors was reported to suppress the development of insulin resistance in diet-induced obesity mouse models (97, 98), and in healthy participants, intravenous infusion of TNF-α was reported to decrease whole-body glucose uptake in insulin clamp tests (99). In a double-blind study, an IL-1 antibody improved postprandial glucose levels (91). IL-6 also reduces the expression of IRS-1 and glucose transporter-4 (100), and administration of IL-6–neutralizing antibodies improved insulin resistance in IkB kinase-β transgenic mice with constitutively activated signaling of NF-k Band hyperglycemia (98). NO interacts with superoxide to form peroxynitrite, which inhibits insulin signaling by s-nitrosylation or nitration of IRS-1, PI3K, and/or Akt (101). Furthermore, inhibition of iNOS or NF-kB decreases insulin resistance in leptin-deficient obese mouse models (100, 102). In the present study, turmeronols A and B and bisacurone significantly inhibited LPS-induced production of these inflammatory mediators in activated macrophages (Supplementary Figure S1). In addition, laboratory and animal studies showed that turmeronols A and B and bisacurone inhibit phosphorylation and nuclear translocation of NF-kB and suppress the mRNA expression of its target genes, including genes encoding IL-1β, IL-6, TNF-α, and iNOS, in various LPS-stimulated cells, including macrophages (24, 45, 103). Bisacurone was also shown to increase the protein expression of peroxisome proliferator-activated receptor alpha, which has anti-inflammatory effects by inhibiting NF-kB activation (104). Therefore, it appears that turmeronols A and B and bisacurone may prevent the production of inflammatory mediators in macrophages by inhibiting the activation of NF-kB and thus improve the exacerbation of insulin signaling associated with chronic inflammation. However, further research is needed to clarify the effects of turmeronols A and B and bisacurone in humans.

## 5 Conclusion

We investigated the effects of CLE and its potential active ingredients turmeronol A, turmeronol B, and bisacurone on chronic inflammation and postprandial glycometabolism. First, we assessed the inhibitory effects on the production of inflammatory mediators in RAW264.7 macrophage cells stimulated with LPS. Then, we performed a 12-week, randomized, double-blind, placebo-controlled study in middle-aged to elderly participants with overweight and glycemia in the normal/prediabetes range. In the *in vitro* study, CLE, turmeronols A and B, and bisacurone significantly inhibited the production of inflammatory mediators in RAW264.7 macrophages stimulated with LPS. In the clinical study, serum levels of hsCRP and HbA1c were significantly lower in the CLE group than in the placebo group, and in the OGTT, the post-load glucose plasma level, time to peak concentrations of glucose and insulin, and insulin sensitivity indices were also significantly improved in the CLE group. These results suggest that daily intake of CLE may have the potential to improve insulin resistance and postprandial hyperglycemia by reducing chronic low-grade inflammation.

## Data availability statement

The original contributions presented in the study are included in the article/Supplementary material, further inquiries can be directed to the corresponding author.

## Ethics statement

The studies involving humans were approved by the institutional review board of Chiyoda Paramedical Care Clinic (Tokyo, Japan). The studies were conducted in accordance with the local legislation and institutional requirements. The participants provided their written informed consent to participate in this study.

## Author contributions

RU: Conceptualization, Data curation, Formal analysis, Investigation, Methodology, Project administration, Visualization, Writing – original draft, Writing – review & editing. CO-H: Conceptualization, Data curation, Investigation, Methodology, Visualization, Writing – original draft, Writing – review & editing. HS: Investigation, Writing – review & editing. RS: Investigation, Writing – review & editing. KM: Conceptualization, Methodology, Project administration, Supervision, Writing – review & editing. SM: Conceptualization, Methodology, Writing – review & editing. YY: Conceptualization, Methodology, Writing – review & editing. YH: Conceptualization, Methodology, Project administration, Supervision, Writing – review & editing.

## Glossary

ANOVA: Analysis of variance
AUC: Area under the curve
BMI: Body mass index
CRP: C-reactive protein
CLE: A mixture of a hot water extract and a supercritical carbon dioxide extract of *Curcuma longa*
FAS: Full analysis set
HbA1c: Hemoglobin A1c
hsCRP: High-sensitive C-reactive protein
IL: Interleukin
iNOS: Inducible nitric oxide synthase
IRS-1: Insulin receptor substrate-1
ITT: Intention to treat
LPS: Lipopolysaccharide
NF-kB: Nuclear factor kappa-light-chain-enhancer of activated B cells
NO: Nitric oxide
OGTT: Oral glucose tolerance test
PPS: Per-protocol set
r-ANOVA: Repeated measures two-way ANOVA
RCT: Randomized, double-blind, placebo-controlled trial
TNF-α: Tumor necrosis factor-alpha
UMIN: University Hospital Medical Information Network
