# Supplementary material for: Curcuma longa extract reduces serum inflammatory markers and postprandial hyperglycemia in healthy but borderline participants with overweight and glycemia in the normal/prediabetes range: a randomized, double-blind, and placebo-controlled trial
Source: Front Nutr. 2024 Jan 29;11:1324196. doi: 10.3389/fnut.2024.1324196 (PMC10859506; doi:10.3389/fnut.2024.1324196)
Supplement: Supplementary file 2 [file Data_Sheet_1.docx]

**Supplementary Materials**


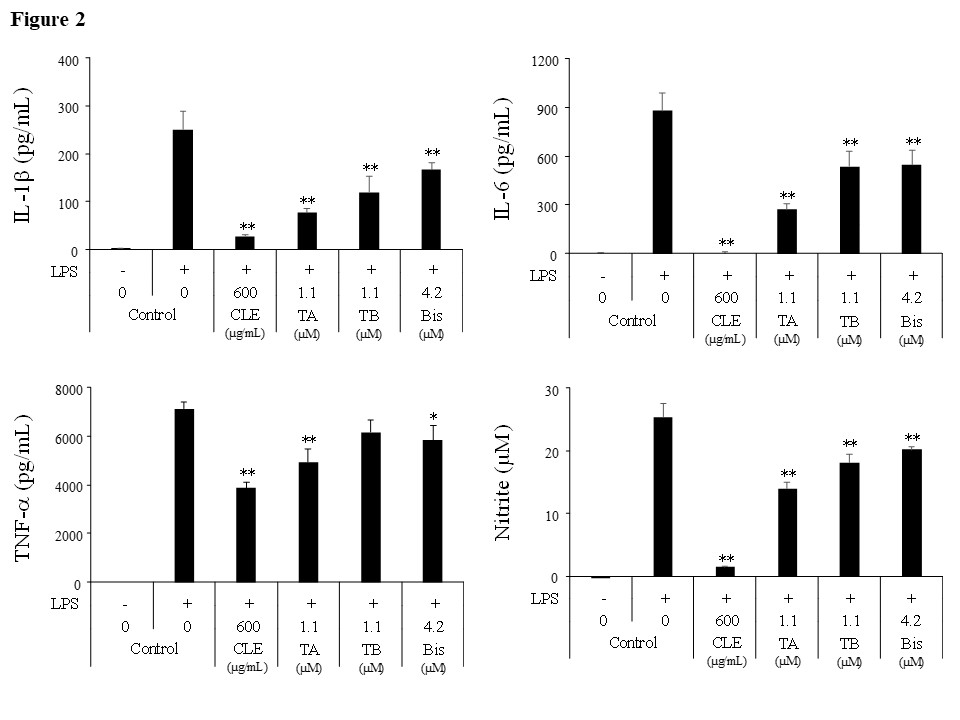


Figure S1. Effects of *Curcuma longa* extract (CLE), turmeronol A, turmeronol B, and bisacurone on the production of inflammatory mediators in RAW264.7 macrophage cells stimulated with lipopolysaccharide (LPS). RAW264.7 macrophage cells were pretreated with CLE, turmeronol A, turmeronol B, and bisacurone for 1 h under serum-free conditions and subsequently stimulated with LPS (200 ng/mL) for 15 h. The levels of interleukin (IL)-1β, IL-6, tumor necrosis factor-α protein, and nitrite (nitric oxide) in culture supernatants were measured by enzyme-linked immunosorbent assay. Data are expressed as means and standard deviations (n = 3). The mean values in the test cells were significantly different from those in the LPS-stimulated control cells. *p < 0.05, **p < 0.01 (Dunnett’s test). Representative results of two or more independent experiments is shown.

Abbreviations: Bis, bisacurone; CLE, a mixture of a hot water extract and a supercritical carbon dioxide extract of *Curcuma longa*; IL, interleukin; LPS, lipopolysaccharide; TA, turmeronol A; TB, turmeronol B; TNF-α, tumor necrosis factor-α
